# Multiple Sclerosis-like Lesions Induced by Radiation: A Case Report and Systematic Review of the Literature

**DOI:** 10.3390/jcm13247554

**Published:** 2024-12-12

**Authors:** Angeliki-Erato Sterpi, Alexandros-Stavros Triantafyllou, Dimitrios Tzanetakos, Eleni Ampantzi, Dimitrios Kitsos, Aikaterini Theodorou, Effrosyni Koutsouraki, Maria Maili, Maria Ioanna Stefanou, Christos Moschovos, Lina Palaiodimou, John Tzartos, Sotirios Giannopoulos, Georgios Tsivgoulis

**Affiliations:** 1 Second Department of Neurology, “Attikon” University Hospital, School of Medicine, National and Kapodistrian University of Athens, 12462 Athens, Greece; angste1995@gmail.com (A.-E.S.); dtzanetakos@med.uoa.gr (D.T.); dkitsos@icloud.com (D.K.); katetheo24@gmail.com (A.T.); sgiannop@uoi.gr (S.G.); 2 First Department of Neurology, AHEPA University Hospital, Aristotle University of Thessaloniki, School of Medicine, 54124 Thessaloniki, Greece

**Keywords:** radiotherapy, radiation-induced neurotoxicity, multiple sclerosis, demyelination, neuro-oncology, magnetic resonance imaging

## Abstract

**Background/Objectives:** Radiotherapy (RT) remains crucial in treating both primary and metastatic central nervous system cancer. Despite advancements in modern techniques that mitigate some toxic adverse effects, magnetic resonance imaging (MRI) scans still reveal a wide range of radiation-induced changes. Radiation can adversely affect neuroglial cells and their precursors, potentially triggering a demyelinating pattern similar to multiple sclerosis (MS). The aim of the current review is to investigate the occurrence and characteristics of such cases presented in the literature. **Methods:** We present the case of a 37-year-old female patient with multiple white matter lesions on a brain MRI, mimicking MS, after the completion of RT sessions. Additionally, a systematic review of the literature (PROSPERO id: CRD42024624053) was performed on 4 January 2024. The databases of MEDLINE and SCOPUS were searched. Case reports or case series of adult patients with white matter lesions in a brain MRI, consistent with the MAGNIMS criteria for MS plaques, after RT, were included in our final synthesis. The PRISMA guidelines were applied. **Results:** The systematic search of the literature revealed 1723 studies, 7 of which conformed to our inclusion criteria, including seven patients in our final analysis. Four of them were female and the mean age was 39 ± 11 years. Several intracranial and extracranial RT types were performed. The symptoms occurred 3 ± 0.8 months after the completion of RT. Lesions were revealed in infratentorial, periventricular and subcortical white matter regions, but not in the spinal cord. All patients who received corticosteroids (83%) showed clinical improvement. Clinical and radiological recurrence occurred in two of the patients during the follow-up period. Fingolimod and Interferon beta-1a were administered to these two patients. **Conclusions:** Radiation-induced demyelination is a critical clinical and radiological entity that requires attention from both oncologists and neurologists. Comprehensive follow-up is essential to identify patients who may benefit from disease-modifying therapies and to distinguish them from those with pre-existing demyelinating conditions.

## 1. Introduction

Brain injury following cranial irradiation is a well-documented adverse effect of radiation therapy (RT), with cognitive decline being its primary association, often described months to years after exposure. Recent efforts have aimed to categorize the side effects of radiation based on the timing of symptom onset: (1) acute (days–weeks), (2) early delayed (1–6 months), and (3) late delayed (>6 months) [1,2]. Symptoms occurring during the initial stages are typically transient and tend to resolve automatically or with the use of corticosteroids, unlike the later stage where the damage is considered permanent.

The incidence of radiation-induced brain injury varies based on several factors, including the total dose, fractionation, and regions irradiated. Research indicates that the occurrence of clinically significant radiation necrosis—an irreversible and severe form of brain injury—ranges according to the total dose, from 5% for lower doses (50.4 Gy) to 10% for higher doses (64.8 Gy) [3]. Additionally, milder forms of brain injury, such as early delayed effects or transient cognitive impairments, are more common but less severe [4]. Radiotherapy-induced demyelination is not extensively quantified in terms of incidence in population-level studies, but it is a recognized adverse effect of cranial radiation.

Acute brain injury emerges days to weeks after exposure and is relatively uncommon with current radiation protocols [1,2]. Patients commonly experience symptoms such as fatigue, headache, nausea, and lethargy. It is primarily associated with endothelial damage and disruption of the blood–brain barrier (BBB), leading to vasogenic edema. While this type of injury often lacks radiological findings, diffuse brain swelling may be observed [1].

**Figure 1:**
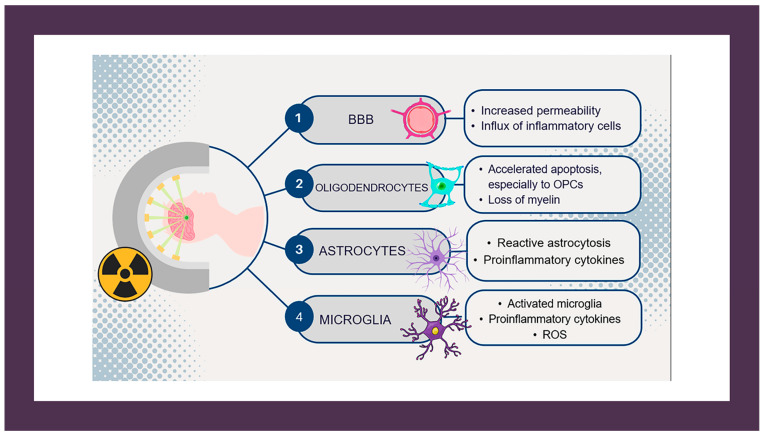
Mechanisms of early delayed CNS injury induced by radiation. Abbreviations: BBB: blood–brain barrier; OPCs: Oligodendrocyte type-2 astrocytes; ROS: reactive oxygen species.

Early delayed injury, typically occurring one to six months post-exposure, is thought to be associated with the transient demyelination of the central nervous system (CNS). Experimental studies indicate that oligodendrocytes—cells responsible for the production of the myelin sheath—are particularly vulnerable to radiation. This results in a significant loss of mature oligodendrocytes and their precursor cells, the oligodendrocyte type-2 astrocytes (OPCs) [5]. Furthermore, radiation-induced neuroinflammation has been implicated in the disruption of the BBB, facilitating the infiltration of peripheral immune cells, cytokines, and toxins (Figure 1). This cascade of events activates glial cells, promoting gliosis and further contributing to CNS injury [6].

During this stage, patients may exhibit symptoms including somnolence, worsening of pre-existing symptoms, temporary cognitive impairment, and new focal deficits. MRI findings can range from non-enhancing white matter hyperintensities on T2-weighted images, to the emergence or enlargement of enhancing lesions near the irradiated tumor region [7].

Late delayed reactions, which manifest several months to years after RT, encompass moderate to severe cognitive impairment attributed to the necrosis of white matter tracts, axonal degeneration, and vascular injury [8,9]. The brain MRI findings are primarily associated with radiation-induced necrosis and leukoencephalopathy, as well as several vascular abnormalities.

In this study, we present the case of a 37-year-old woman who underwent intensity-modified radiotherapy (IMRT) for metastatic breast cancer. Subsequently, she developed ataxia with multiple sclerosis (MS)-like lesions, which were detected on brain MRI. Furthermore, we conducted a systematic literature review to identify cases with similar clinical and radiological manifestations following RT.

This study offers important contributions to the existing body of literature by presenting a relatively rare case of radiation-induced demyelination in a patient without a known preexisting autoimmune disorder. The occurrence of radiation-induced demyelination is infrequent, or quite under-recognized. Our investigation not only seeks to elucidate the underlying pathophysiological mechanisms but also provides further insights into the disease’s progression. Moreover, the findings of this study are particularly valuable for clinicians involved in the management of patients receiving RT, as they provide critical information to aid in the recognition and management of potential neurological complications associated with this treatment.

## 2. Methods

### 2.1. Case Report

We report the case of a patient with multiple demyelinating lesions on a brain MRI, mimicking MS, after the completion of IMRT sessions; the MRI findings were initially attributed to brain metastases. A written informed consent form was signed by the patient for participation and publication of this study.

### 2.2. Systematic Review of the Literature

A systematic search of the literature was performed to identify available observational studies regarding adult patients with multiple white matter lesions (WMLs), mimicking demyelinating disorders, after receiving RT. The characteristics of the lesions included a round or ovoid shape, high signal intensity in T2-weighted MR images, a diameter of a few millimeters up to approximately 1 cm and white matter localization (especially in the periventricular or the juxtacortical region) [10,11]. Two reviewers (AES, AST) performed independently the literature search. We searched the databases of MEDLINE and SCOPUS, using the following terms: “demyelination”, “demyelinating”, “white matter lesion”, “radio”, “radiotherapy”, “radiation”, “radiation therapy”, “radiation treatment”, “actinotherapy”, and “irradiation”. No search filters were applied. The search of the literature was performed on 4 January 2024. The reference lists of the articles included in the qualitative synthesis were also reviewed.

Initially, we screened the titles and the abstracts in order to determine which studies could meet the inclusion criteria. Editorial comments, letters to the editor, reviews, original research lacking primary data, animal studies, and histopathological studies were excluded. Afterwards, the full-text articles were thoroughly assessed using the PICOS (population, intervention, comparison, outcome, study design) criteria [12,13]. The PICOS criteria were set as follows: (P) adult patients; (I) RT; (C) none; (O) CNS demyelinating lesions; (S) observational studies, including case series and case reports.

Published articles with lack of brain MRI scan data were rejected. Cases with a history of previous demyelinating disease or demyelinating lesions on previous MRI were also excluded. Additionally, case reports of diffuse leukoencephalopathy or solitary lesions following RT were rejected. All eligible studies were independently evaluated by two reviewers (AES, AST). All controversies were unraveled after discussion with a third evaluator (DT).

According to our knowledge, no other systematic reviews regarding demyelinating lesions after RT exist in the literature. We adhered to the PRISMA guidelines and used the PICOS criteria, according to the grade methodology [12,13,14]. A complete PRISMA checklist is provided as a supplementary file, showing where each item is addressed (Supplementary Table S1). Our study group is very experienced in conducting systematic reviews as shown by our previous studies [15,16]. The protocol for the present study was submitted to PROSPERO (CRD42024619314).

## 3. Results

### 3.1. Case Report

A 37-year-old woman presented to the Neurology Department with a two-month history of progressively worsening gait instability and limb ataxia. Her medical history included BRCA1-positive breast cancer diagnosed three years prior, accompanied by lung metastases. Initial management consisted of surgical resection of the primary tumor and lung metastases, followed by adjuvant hormonal and targeted chemotherapy with Triptorelin, Palbociclib, and Letrozole, resulting in clinical stability for two years.

The disease subsequently recurred with new pulmonary metastases, necessitating a shift in therapy to Bevacizumab, an anti-vascular endothelial growth factor (VEGF) agent, combined with Paclitaxel. Over the next six months, the disease further progressed, with additional metastatic lesions identified in the liver and thoracolumbar spine.

**Figure 2:**
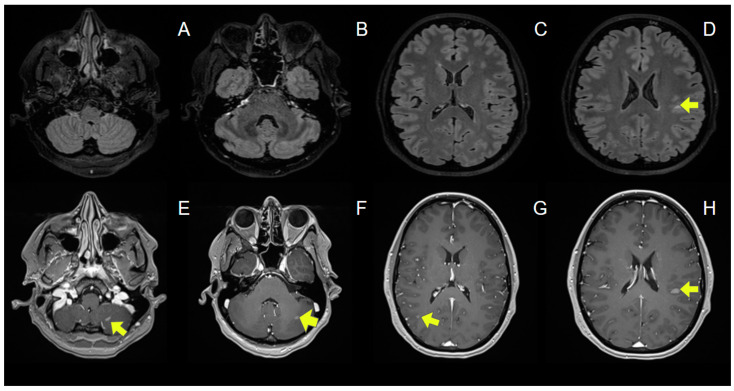
Brain MRI prior to radiation therapy. Metastatic lesions in the gray–white matter junction are indicated by arrows on axial FLAIR (**D**) and axial post-gadolinium T1-MPRAGE images (**E**–**H**). (**A**–**H**). Abbreviations: FLAIR: fluid-attenuated inversion recover; MPRAGE: magnetization prepared rapid gradient echo.

A comprehensive reassessment, including a full-body CT scan, was performed to guide further treatment planning. Incidentally, the CT scan revealed presumed brain metastases, despite the absence of neurological symptoms at that time. A follow-up brain MRI confirmed multiple nodular lesions, predominantly located at the gray–white matter junction, with nodular enhancement (Figure 2). A spinal MRI showed no evidence of spinal cord involvement but confirmed osseous metastases in the thoracolumbar spine. Cerebrospinal fluid (CSF) cytology obtained via lumbar puncture demonstrated the presence of malignant cells, supporting the diagnosis of brain metastases.

In light of these findings, the therapeutic regimen was adjusted to include Olaparib, a poly (ADP-ribose) polymerase (PARP) inhibitor, specifically targeting BRCA1-mutated cancer. Additionally, the patient underwent 10 sessions of IMRT to the brain, delivering a total dose of 30 Gy. The new treatment regimen resulted in the stabilization of her metastatic disease, with residual metastases in the liver, lungs, and bones, but with a reduction in size.

On neurological examination, the most prominent findings were a wide-based gait, incoordination, upper limb dysmetria, and intention tremor. Aside from these cerebellar signs, her muscle strength, tone, and sensory examination were within normal limits, with deep tendon reflexes also intact, and her plantar responses were flexor.

**Figure 3:**
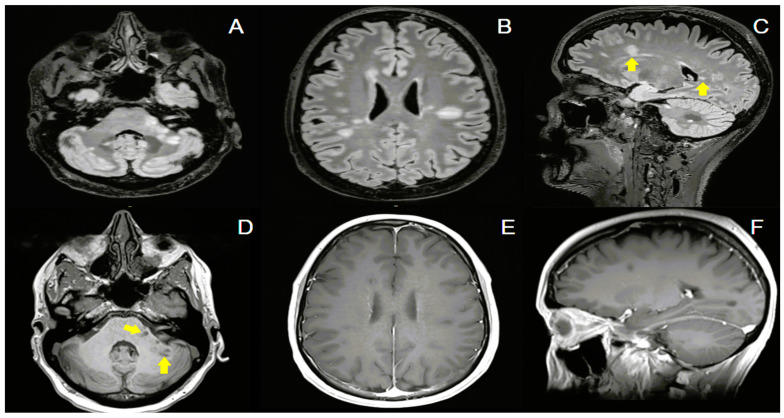
Brain MRI following radiation therapy. FLAIR images showed several hyperintense ovoid lesions in both infratentorial (**A**) and supratentorial regions (periventricular and subcortical/juxtacortical) (**B**,**C**). Several of these lesions are oriented perpendicular to the long axis of the lateral ventricles ((**C**), arrows). A few hypointense lesions on T1-MPRAGE images, consistent with “black holes”, are depicted in image (**D**) (arrows). On T1-weighted post-contrast imaging (**E**,**F**), there is no evidence of gadolinium enhancement. Abbreviations: FLAIR: fluid-attenuated inversion recover; MPRAGE: magnetization prepared rapid gradient echo.

A follow-up brain MRI revealed multiple new hyperintense lesions on FLAIR and T2-weighted images without contrast enhancement (Figure 3). These lesions were ovoid in shape and distributed across both supratentorial (periventricular and juxtacortical) and infratentorial (brainstem and cerebellar hemispheres) regions, in close proximity to the field of radiation treatment. The imaging characteristics of these lesions were consistent with the MAGNIMS criteria for MS plaques [11]. Additionally, non-enhancing T1 hypointense lesions, commonly referred to as “black holes”, were identified, indicative of chronic axonal loss. However, these lesions are not considered adjunctive criteria for fulfilling the dissemination in time (DIT) requirement according to the 2017 McDonald MS criteria [17].

In order to differentiate CNS demyelination, a lumbar puncture was performed, revealing no abnormalities other than slightly elevated protein without evidence of IgG production or CSF-specific oligoclonal bands (OCBs) (white blood cell count: 0 cells/mm^3^, protein: 55.9 mg/dL, CSF glucose/serum glucose: 61%, CSF IgG index: 0.556, CSF cytology: negative).

A workup for other systemic autoimmune diseases was conducted and disclosed no abnormal findings. Additionally, an MRI of the cervical and thoracic spinal cord was performed, which did not reveal any spinal cord lesion.

The patient received treatment with 1000 mg of Methylprednisolone daily for a total of 5 days, with partial improvement in gait unsteadiness. Consequently, she was placed on oral Methylprednisolone 64 mg per day with gradual tapering over 3 weeks.

Since there was not enough evidence to support the DIT criterion and it was not possible to exclude that the lesions were radiotherapy-induced, the diagnosis of MS was not established. No other disease-modifying therapy was proposed, and a recommendation was made for MRI surveillance. The patient was followed up for six months without any evidence of new neurological symptoms or new brain lesions.

### 3.2. Systematic Review of the Literature

**Figure 4:**
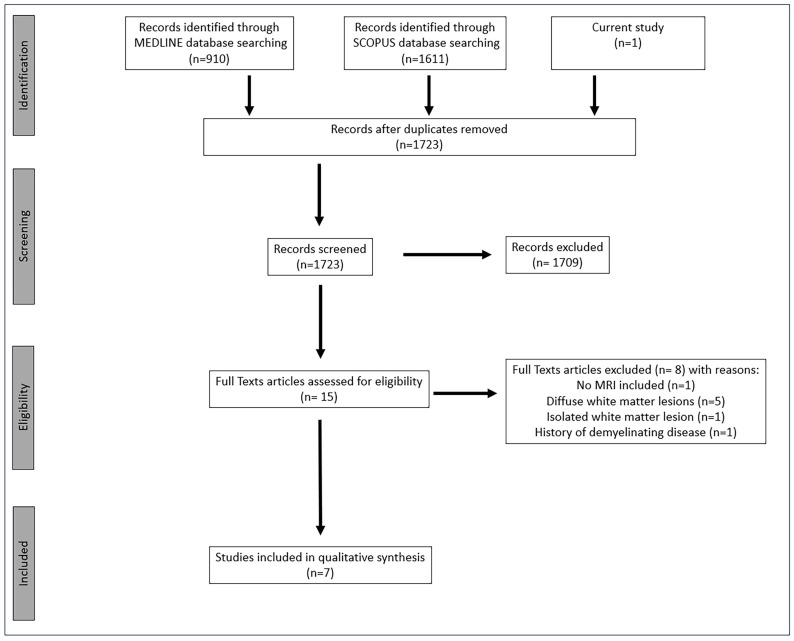
Flowchart of systematic review. Abbreviations: n: number.

The systematic search of the MEDLINE and SCOPUS databases revealed a total of 910 and 1611 records, respectively (Figure 4). After the duplicates’ exclusion, 1723 studies were assessed. A total of 1709 papers were excluded based on their irrelevance to the topic or their classification as letters to the editor, review articles, editorial comments, animal studies, histopathological studies, or original research lacking primary data, as assessed through initial screening of title or abstract. After the duplicates’ exclusion and the initial screening, we evaluated the full text of 15 studies, 8 of which were further rejected. These studies were excluded due to evidence of an isolated lesion [18] or diffuse white matter lesions [19,20,21,22,23] on brain imaging, prior history of demyelinating disease [24] or absence of MRI images [25]. Therefore, we selected seven eligible studies for inclusion in the qualitative synthesis [26,27,28,29,30,31].

Four female and three male patients fulfilling our criteria were identified; their main characteristics are summarized in the Table 1. The age of these patients ranged between 28 and 65 years old (mean age 39 ± 11 years). They had received RT due to primary CNS tumors (58%), brain metastases (14%), nasopharyngeal carcinoma (14%), and trigeminal neuralgia (14%). The types of the RT performed included IMRT (43%), stereotactic radiosurgery (SRS) (29%), whole ventricular radiation therapy (WVRT) (14%), and RT targeted on extracranial structures (pharynx and neck lymph nodes) (14%). The total radiation dose received ranged between 24 and 90 Gy (mean radiation dose 51.6 ± 20.4 Gy). Chemotherapy was offered to 43% of the patients in a period ranging between 2 and 24 months prior to the symptoms’ onset. The chemotherapeutic agents used included Paclitaxel, Carboplatin, Etoposide, Ifosfamide, Cisplatin, and Fluorouracil.

The neurological symptoms developed during a period ranging between 2 and 4 months (mean 3 ± 0.8 months) after the completion of RT, while the most common neurological symptoms included ataxia, sensory symptoms, diplopia, and nystagmus. The brain MRI disclosed multiple WMLs affecting mostly the brainstem, the cerebellum, the periventricular, and the subcortical white matter in a descending frequency. Most of the WMLs showed contrast enhancement (83% of cases), in a nodular or ring enhancing pattern. No lesions were detected in the spinal cord. Intrathecal OCBs synthesis occurred in 50% of the patients.

Regarding treatment, 86% of the patients received corticosteroids (Methylprednisolone, Prednisolone, Dexamethasone) intravenously or per os, with or without tapering. All patients showed clinical improvement, as well as the one who did not receive any treatment; however, this patient experienced no focal neurological signs, but only subjective cognitive impairment. The follow-up period ranged between 6 and 24 months (mean 11.6 ± 5.6 months). Two out of seven patients developed clinical and radiological recurrence at 6 and 10 months after the onset of the first episode, respectively. They received disease modifying therapies with Fingolimod and Interferon beta-1a, respectively.

## 4. Discussion

Herein, we present the case of a young female patient with known metastatic breast cancer, who developed multiple demyelinating brain lesions, two months after the completion of RT for presumed brain metastases. We also conducted a systematic review of the literature, in search of similar cases. These cases are remarkable due to their CNS neuroimaging similarities with MS, as the newly presented lesions conform to the MAGNIMS criteria, including being more than 3 mm in diameter, ovoid, perpendicular to the ventricles, and located both supra- and infratentorially [11].

Interestingly, the sex ratio of the patients described above was approximately 1:1, in contrast to the female predominance among the MS patients [32]. The mean age of onset was also higher compared to MS patients. This may be explained by the higher incidence of cancer in older age. Furthermore, intrathecal OCB production in the aforementioned patients was demonstrated in lower frequency (3/7) compared to MS patients [33]. It is worth mentioning that in our review, only one-third of the cases (2/7) with demyelinating lesions after RT showed clinical and imaging recurrence. Both of these cases were positive for intrathecal expression of OCBs and underwent SRS.

The WMLs and the clinical history of the patients described in this study may be explained by one of the following two hypotheses: Early delayed radiation-induced demyelination or MS triggered by RT in predisposed patients. Focal neurological deficits and demyelinating lesions may occur one to six months after the completion of RT (early delayed radiation injury) [1,3]. The symptoms are usually reversible, and corticosteroids may contribute to their alleviation. This was also manifested in the case described by our group. This phenomenon is thought to result from a disruption of the BBB, accompanied by an influx of inflammatory cells, activation of microglia, and radiation-induced toxicity to oligodendrocytes. These processes initiate an inflammatory cascade that leads to demyelination, and if unresolved, can progress to gliosis and leukoencephalopathy in the CNS. This proposed pathophysiological mechanism closely resembles that of MS, where disruption of the BBB permits activated T-cells to attack the myelin sheath, resulting in damage to oligodendrocytes. Microglia contributes by supporting the inflammatory response, further exacerbating neuronal damage.

On the other hand, RT may act as an environmental trigger for the manifestation of MS in susceptible patients [26]. The induction of oligodendrocyte apoptosis and the subsequent release of multiple antigenic substances can elicit a secondary autoimmune response, which, in predisposed individuals, may activate a pathophysiological cascade analogous to the mechanisms underlying MS. This hypothesis is further substantiated by our study, in which two patients, both of whom were positive for OCBs, experienced a relapse several months after their initial presentation and were subsequently managed with immunomodulatory therapy for MS [28,29]. Additionally, Milic et al. have presented two patients with a history of demyelinating disease, who suffered from a relapse after the completion of RT, further reinforcing this hypothesis [24].

Increased total radiation dosage and brain volume irradiated are usually associated with a greater clinical and imaging effect [34]. The RT techniques utilized in the patients detected through our search comprised a variety of methods: IMRT and SRS, which precisely target specific areas within the brain; WVRT, which impacts broader regions of the brain; and RT, which was directed towards areas adjacent to the CNS (pharynx and neck lymph nodes). The RT dose varied between 24 and 90 Gy. Regardless of the target specificity or dose of these techniques, WMLs were detected even in remote brain areas.

The administration of chemotherapy in 42% of the cases detected by our literature search may be noted as a confounding factor. However, the substances used are more commonly associated with ischemic stroke (Cisplatin), reversible cerebral vasoconstriction syndrome (Ifosfamide) and leukoencephalopathy (5-Fluorouracil) (rather than multiple demyelinating CNS lesions), usually demonstrated acutely after the chemotherapy administration [35,36,37]. The mean duration between the chemotherapy delivery and the symptoms’ onset of the patients detected by our search was approximately 10 months. Therefore, chemotherapy may not be the culprit behind the clinical and imaging progression of these patients.

Another limitation of our study is the relatively small number of cases detected after the systematic review of the literature. Due to this limitation, our results must be reviewed with caution. However, in order to detect as many cases similar to ours as possible, we systematically searched two databases (MEDLINE, SCOPUS).

Moreover, it is worth noting that the 6-month follow-up for our patient is relatively short, and the absence of recurrence does not rule out the possibility of a future MS diagnosis.

In conclusion, oncologists and neurologists should be aware of the phenomenon of CNS demyelination after RT, as this therapy is administered widely among cancer patients. The prolonged monitoring and further presentation of similar cases in the literature may provide useful information to attain safer conclusions regarding treatment and management.

## Figures and Tables

**Table 1:** Patients’ characteristics in seven cases included in the current systematic review.

Reference	Age/Sex	Initial Diagnosis	Medical History	Social/ Family History	Medi- Cation (Last 2 Years)	RT Type	Total RT Dose (Gy)	Total RT Duration (Days)	Onset of Symptoms After RT	Symptoms	CSF Studies	CSF Specific OCBs	Infection Panel	Auto- Immunity Panel	Regions Affected	Gd +	Treatment	Clinical Response	Recurrence (Time After Onset—Symptoms)	Imaging Follow-Up (Time After Onset)	Treatment After Recurrence
**Sterpi AE, et al. (2024)**	37/F	Breast Cancer	No	No	OLP, PLB, TRP, BVZ, LTR	IMRT	30	10	2 months	Ataxia	Normal	No	Negative	Negative	Periventricular WM, corpus callosum, brainstem, cerebellum	-	IVMP with gradual tapering	Yes	No (6 months)	No new lesions (6 months)	N/A
**Wong OY, et al. (2019) [31]**	31/F	Pituitary Macroadenoma	No	N/A	CBR	IMRT	50.4	28	4 months	Decreased visual acuity, diplopia, facial numbness, tongue hemianesthesia	Normal	No	N/A	N/A	Subcortical WM, periventricular WM, brainstem, cerebellum	N/A	PSL	Yes	No (12 months)	No new lesions (12 months)	N/A
**Borges A, et al. (2021) [26]**	28/M	Suprasellar Germinoma	No	No	CRB, ETP, IFS	WVRT	24	15	4 months	Subjective cognitive impairment	Normal	Yes	Negative	Negative	Periventricular WM, subcortical WM, brainstem, cerebellum	+	No	Yes	No (12 months)	No new lesions (10 months)	N/A
**Kemp S, et al. (2016) [29]**	65/F	Trigeminal Neuralgia	N/A	N/A	GBP	SRS	90	1	3 months	Facial numbness, hemiparaesthesia, ataxia	Normal	Yes	N/A	N/A	TREZ	+	IVMP	Yes	Yes (10 months—left lower limb weakness, gait ataxia)	Periventricular WM, cervical spinal cord (10 months)	Fingolimod
**Guillemin F, et al. (2020) [28]**	36/F	Pituitary Macroadenoma	No	N/A	N/A	SRS	50.4	28	3 months	Dizziness, ataxia, nystagmus, left arm hypoesthesia	Normal	Yes	Negative	Negative	Brainstem, cerebellum, subcortical WM	+	IVMP	Yes	Yes (6 months—weakness Nystagmus)	Infratentorial lesions (6 months)	Beta-1A- Interferon
**Esakia T, et al. (2021) [27]**	34/M	Nasophary-ngeal Carcinoma	No	N/A	CSP, 5FU	Focal RT	66	33	2 months	Headache, ataxia, nystagmus	N/A	N/A	N/A	N/A	Cerebellum, periventricular WM	+	IVDEX with gradual tapering	Yes	No (24 months)	No new lesions (24 months)	N/A
**Toljan K, et al. (2021) [30]**	41/M	Pituitary Macroade- noma	No	No	N/A	IMRT	50.4	28	3 months	Diplopia, facial hemihypoesthesia, dysarthria, tongue numbness, ataxia, hemiparesis, single-sided hypoacusia	13 cells/μL Protein: 50 mg/dL	No	Negative	Negative	Brainstem, cerebellum	+	IVMP followed by DEX	Yes	No (11 months)	No new lesions (11 months)	N/A

Abbreviations: 5FU: 5-Fluorouracil; BVZ: Bevacizumab; CBR: Cabergoline; CRB: Carboplatin; CSF: cerebrospinal fluid; CSP: Cisplatin; DEX: Dexamethasone; ETP: etoposide; F: female; GBP: Gabapentin; Gd+: Gadolinium enhancement; IFS: Ifosfamide; IMRT: intensity-modulated radiation therapy; IVDEX: intravenous Dexamethasone; IVMP: intravenous Methylprednisolone; LTR: Letrozole; M: male; N/A: not applicable; OCBs: oligoclonal bands; OLP: Olaparib; PLB: Palbociclib; PSL: Prednisolone; RT: radiation therapy; SRS: stereotactic radiosurgery; TREZ: trigeminal root entry zone; TRP: Triptorelin; WM: white matter; WVRT: whole ventricular radiation therapy.

## Data Availability

All data needed to evaluate the conclusions in the paper are presented in the main manuscript. Additional data related to this paper may be requested from the corresponding author, upon reasonable request.

## Supplementary Materials

The following supporting information can be downloaded at: https://www.mdpi.com/article/10.3390/jcm13247554/s1, Table S1: PRISMA 2020 Checklist [12].

## References

[1] Tofilon P.J., Fike J.R. (2000). The radioresponse of the central nervous system: A dynamic process. Radiat. Res..

[2] Wu B., Li S., Wang J., Qiu W., Gao H. (2023). Bibliometric and visualization analysis of radiation brain injury from 2003 to 2023. Front. Neurol..

[3] Greene-Schloesser D., Robbins M.E., Peiffer A.M., Shaw E.G., Wheeler K.T., Chan M.D. (2012). Radiation-induced brain injury: A review. Front. Oncol..

[4] Jacob J., Durand T., Feuvret L., Mazeron J.-J., Delattre J.-Y., Hoang-Xuan K., Psimaras D., Douzane H., Ribeiro M., Capelle L. (2018). Cognitive impairment and morphological changes after radiation therapy in brain tumors: A review. Radiother. Oncol..

[5] Van Der Maazen R.W., Kleiboer B.J., Verhagen I., Van Der Kogel A. (1993). Repair Capacity of Adult Rat Glial Progenitor Cells Determined by an In Vitro Clonogenic Assay after In Vitro or In Vivo Fractionated Irradiation. Int. J. Radiat. Biol..

[6] Nakkazi A., Forster D., Whitfield G.A., Dyer D.P., Dickie B.R. (2024). A systematic review of normal tissue neurovascular unit damage following brain irradiation—Factors affecting damage severity and timing of effects. Neuro-Oncol. Adv..

[7] Katsura M., Sato J., Akahane M., Furuta T., Mori H., Abe O. (2021). Recognizing Radiation-induced Changes in the Central Nervous System: Where to Look and What to Look For. RadioGraphics.

[8] Valk P.E., Dillon W.P. (1991). Radiation injury of the brain. AJNR Am. J. Neuroradiol..

[9] Zhong X., Huang B., Feng J., Yang W., Liu H. (2015). Delayed leukoencephalopathy of non-small cell lung cancer patients with brain metastases underwent whole brain radiation therapy. J. Neuro-Oncol..

[10] Barkhof F., Koeller K.K., Hodler J., Kubik-Huch R.A., von Schulthess G.K. (2020). Demyelinating Diseases of the CNS (Brain and Spine). Diseases of the Brain, Head and Neck, Spine 2020–2023: Diagnostic Imaging.

[11] Wattjes M.P., Ciccarelli O., Reich D.S., Banwell B., de Stefano N., Enzinger C., Fazekas F., Filippi M., Frederiksen J., Gasperini C. (2021). 2021 MAGNIMS–CMSC–NAIMS consensus recommendations on the use of MRI in patients with multiple sclerosis. Lancet Neurol..

[12] Page M.J., McKenzie J.E., Bossuyt P.M., Boutron I., Hoffmann T.C., Mulrow C.D., Shamseer L., Tetzlaff J.M., Moher D. (2021). Updating guidance for reporting systematic reviews: Development of the PRISMA 2020 statement. J. Clin. Epidemiol..

[13] Higgins J.P., Green S. (2013). Cochrane Handbook for Systematic Reviews of Interventions, *Version 5.1.0*.

[14] Guyatt G.H., Oxman A.D., Vist G.E., Kunz R., Falck-Ytter Y., Alonso-Coello P., Schünemann H.J. (2008). GRADE: An emerging consensus on rating quality of evidence and strength of recommendations. BMJ.

[15] Giannopapas V., Kitsos D., Tsogka A., Tzartos J.S., Paraskevas G., Tsivgoulis G., Voumvourakis K., Giannopoulos S., Bakalidou D. (2023). Sexual dysfunction therapeutic approaches in patients with multiple sclerosis: A systematic review. Neurol. Sci..

[16] Stefanou M.-I., Giannopapas V., Kitsos D.K., Chondrogianni M., Theodorou A., Kosmidou M., Vlotinou P., Bakirtzis C., Andreadou E., Tzartos J.S. (2024). Prevalence and epidemiology of stroke in patients with multiple sclerosis: A systematic review and meta-analysis. J. Neurol..

[17] Thompson A.J., Banwell B.L., Barkhof F., Carroll W.M., Coetzee T., Comi G., Correale J., Fazekas F., Filippi M., Freedman M.S. (2018). Diagnosis of multiple sclerosis: 2017 revisions of the McDonald criteria. Lancet Neurol..

[18] Kihlstrom L., Guo W.-Y., Karlsson B., Lindquist C., Lindqvist M. (1997). Magnetic resonance imaging of obliterated arteriovenous malformations up to 23 years after radiosurgery. J. Neurosurg..

[19] Omuro A.M., Ben-Porat L.S., Panageas K.S., Kim A.K., Correa D.D., Yahalom J., DeAngelis L.M., Abrey L.E. (2005). Delayed neurotoxicity in primary central nervous system lymphoma. Arch. Neurol..

[20] Redjal N., Agarwalla P.K., Dietrich J., Dinevski N., Stemmer-Rachamimov A., Nahed B.V., Loeffler J.S. (2015). Remote acute demyelination after focal proton radiation therapy for optic nerve meningioma. J. Clin. Neurosci..

[21] Lai R., Abrey L.E., Rosenblum M.K., DeAngelis L.M. (2004). Treatment-induced leukoencephalopathy in primary CNS lymphoma: A clinical and autopsy study. Neurology.

[22] Tsuruda J.S., Kortman K.E., Bradley W.G., Wheeler D., Van Dalsem W., Bradley T., Tsuruda K.K.J., Curnes J., Laster D., Ball (1987). Radiation effects on cerebral white matter: MR evaluation. Am. J. Roentgenol..

[23] Zhong G., Zhang J., Liu X., Yang S.B., Gu H.B. (2022). Astrocytoma with myelin oligodendrocyte glycoprotein antibody associated encephalomyelitis: A case report. Medicine.

[24] Milic M., Rees J.H. (2017). Acute demyelination following radiotherapy for glioma: A cautionary tale. Pract. Neurol..

[25] Becker M., Schroth G., Zbaren P., Delavelle J., Greiner R., Vock P., Allal A., Rüfenacht D.A., Terrier F. (1997). Long-term changes induced by high-dose irradiation of the head and neck region: Imaging findings. RadioGraphics.

[26] Borges A., Garcez D., Pedro C., Passos J. (2021). Chemoradiation induced multiple sclerosis-like demyelination. eNeurologicalSci.

[27] Esakia T., Antia T., Janelidze M., Mariamidze A., Okujava M. (2021). Acute Disseminated Encephalomyelitis Following Chemoradiotherapy in an Adult Patient with Nasopharyngeal Cancer. Cureus.

[28] Guillemin F., Biau J., Conde S., Clavelou P., Dupic G. (2020). Multiple sclerosis as differential diagnosis of radionecrosis for post-irradiation brain lesions: A case report. Clin. Transl. Radiat. Oncol..

[29] Kemp S., Allan R.S., Patanjali N., Barnett M., Jonker B. (2016). Neurological deficit following stereotactic radiosurgery for trigeminal neuralgia. J. Clin. Neurosci..

[30] Toljan K., Kshettry V.R., Chao S.T. (2021). Delayed Radiation-Therapy-Induced Cerebral Demyelination. Appl. Radiat. Oncol..

[31] Wong O.Y., Tham Y.-H., Lai M., Davagnanam I., Bremner F. (2019). Radiation-Induced Multiphasic Demyelination. J. Neuro-Ophthalmol..

[32] Oh J., Vidal-Jordana A., Montalban X. (2018). Multiple sclerosis: Clinical aspects. Curr. Opin. Neurol..

[33] Andersson M., Alvarez-Cermeno J., Bernardi G., Cogato I., Fredman P., Frederiksen J., Fredrikson S., Gallo P., Grimaldi L.M., Gronning M. (1994). Cerebrospinal fluid in the diagnosis of multiple sclerosis: A consensus report. J. Neurol. Neurosurg. Psychiatry.

[34] Tanguturi S.K., Alexander B.M. (2018). Neurologic Complications of Radiation Therapy. Neurol. Clin..

[35] Newton H.B. (2012). Neurological complications of chemotherapy to the central nervous system. Handb. Clin. Neurol..

[36] Ali Mohamed D., Semedo A., Adeyemi B., Hessissen L., El Kababri M., Allali N., Chat L., El Haddad S. (2021). Reversible Encepahlopathy Induced by Ifosfamide with Brain Imaging. Glob. Pediatr. Health.

[37] Hemachudha P., Rattanawong W., Pongpitakmetha T., Phuenpathom W. (2023). Fluorouracil-induced leukoencephalopathy mimicking neuroleptic malignant syndrome: A case report. J. Med. Case Rep..

